# Fetus in Fetu: A Rare Cause of an Abdominal Lump

**DOI:** 10.7759/cureus.71464

**Published:** 2024-10-14

**Authors:** Debiprasad Sahoo, Nitin Borle, Sachin Suryawanshi, Rajendra Habib, Meenakshi Balasubramanian

**Affiliations:** 1 General Surgery, Topiwala National Medical College and Bai Yamunabai Laxman Nair Charitable Hospital, Mumbai, IND; 2 Pathology, Topiwala National Medical College and Bai Yamunabai Laxman Nair Charitable Hospital, Mumbai, IND

**Keywords:** adult teratoma, diagnostic and therapeutic challenge, embryonic germ layers, explorative laparotomy, fetus in fetu, lump in the abdomen, parasitic twin, retroperitoneal tumor

## Abstract

Fetus in fetu (FIF) is a rare anomaly characterized by the presence of a monozygotic twin that becomes incorporated into the abdomen of its sibling during fetal development. FIF is a rare congenital condition typically identified in infancy. We present a case of FIF in a 16-year-old male, which is an extremely rare presentation during adolescence. Teratoma is one of the differentials; however, FIF can be distinguished from teratoma by the presence of a vertebral column, often accompanied by a proper arrangement of other organs or limbs around it. Computed tomography (CT) imaging preoperatively diagnosed the FIF, prompting the decision for exploratory laparotomy. Histopathological analysis confirmed the diagnosis of FIF. Surgical excision is considered the optimal treatment.

## Introduction

Fetus in fetu (FIF) is an uncommon developmental anomaly where a mass of tissue resembling a fetus develops inside the body of its twin. The phenomenon was first described by Young [1] in 1808. It is estimated to occur in approximately one in 500,000 live births [2]. Most cases have been documented in neonates and children, with only nine cases reported in individuals older than 15 years. We present a case of FIF in a 16-year-old male.

## Case presentation

A 16-year-old male presented with an abdominal lump first noticed 10 years ago. The lump was insidious in onset and gradually increased over a period. It was associated with occasional, intermittent abdominal pain. There was no history of loss of appetite, loss of weight, or urinary symptoms. General, physical, and systemic examinations were all within normal limits. Abdominal examination revealed approximately 10×10 cm mass in the left lumbar region. The mass was firm and non-tender, with a regular surface and well-defined edges. It was freely mobile and non-pulsatile.

**Figure 1:**
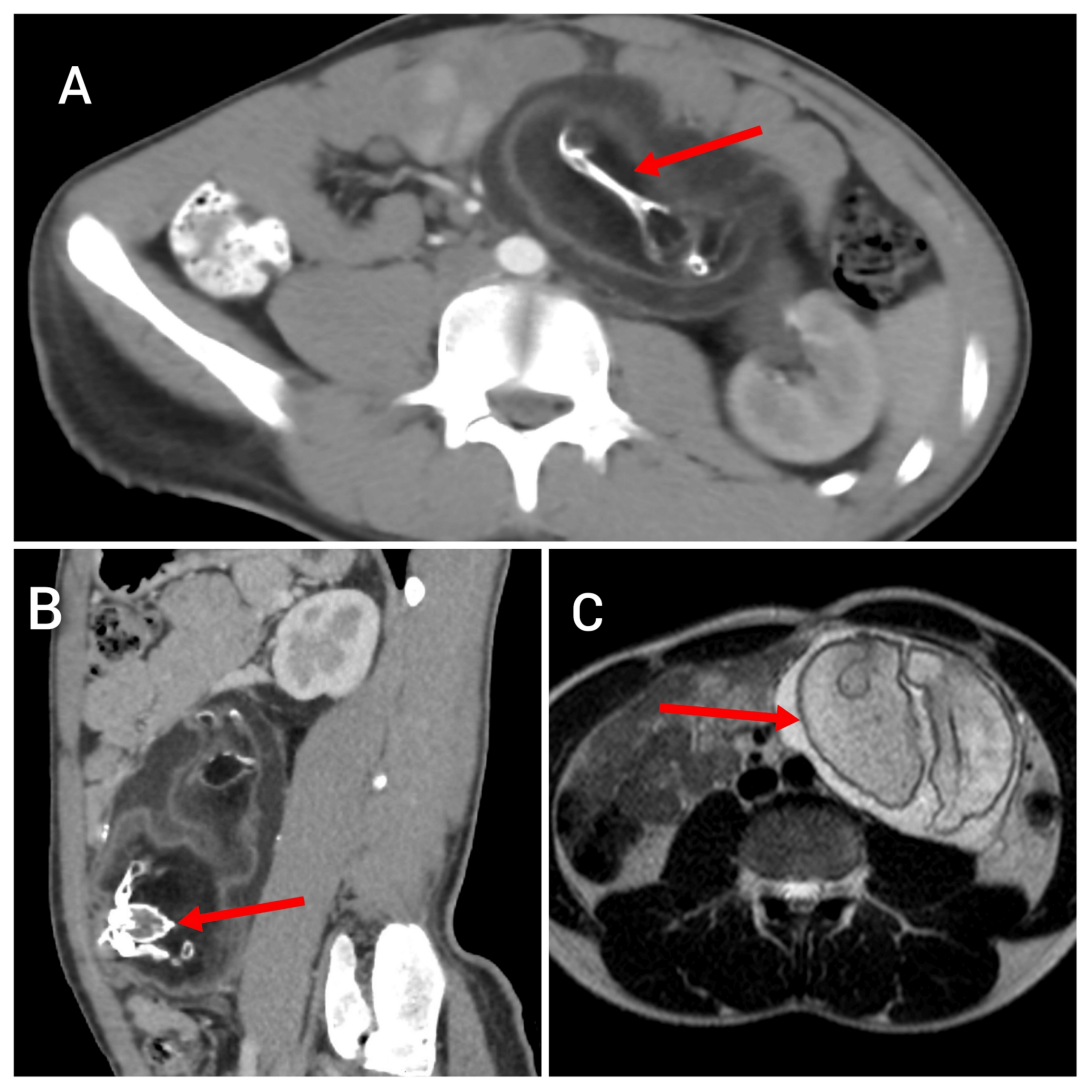
(A) Axial section and (B) sagittal section of CT. (C) Axial section of MRI (A) The arrow indicates an axial section of a CT scan with a long bone-like structure. (B) The arrow points to a sagittal section showing a structure resembling vertebrae. (C) The arrow highlights an MRI scan showing a lesion in the left lumbar region containing both fatty and soft tissue components. CT: computed tomography; MRI: magnetic resonance imaging

Contrast-enhanced computed tomography (CECT) scan revealed a lesion measuring 13.7×9.6 cm in the left lumbar region, posterior to the lower pole of the left kidney. The lesion abutted the left ureter and caused compression at the mid-ureteric level. It exhibited vertebrae-like structures and calcification. Poor enhancement was observed post-contrast, suggesting a possible FIF diagnosis. On magnetic resonance imaging (MRI), a lesion in the left lumbar region was noted containing hypodense fatty and soft tissue components along with calcifications of various sizes and shapes, resembling the shape of vertebrae and long bones. The fat plane with surrounding organs was preserved. Tumor markers including serum alpha-fetoprotein (AFP) and beta subunit of human chorionic gonadotropin (β-HCG) as well as routine blood investigations were all within normal limits (Figure 1).

Based on the clinical and imaging findings, a preoperative diagnosis of FIF was made.

**Figure 2:**
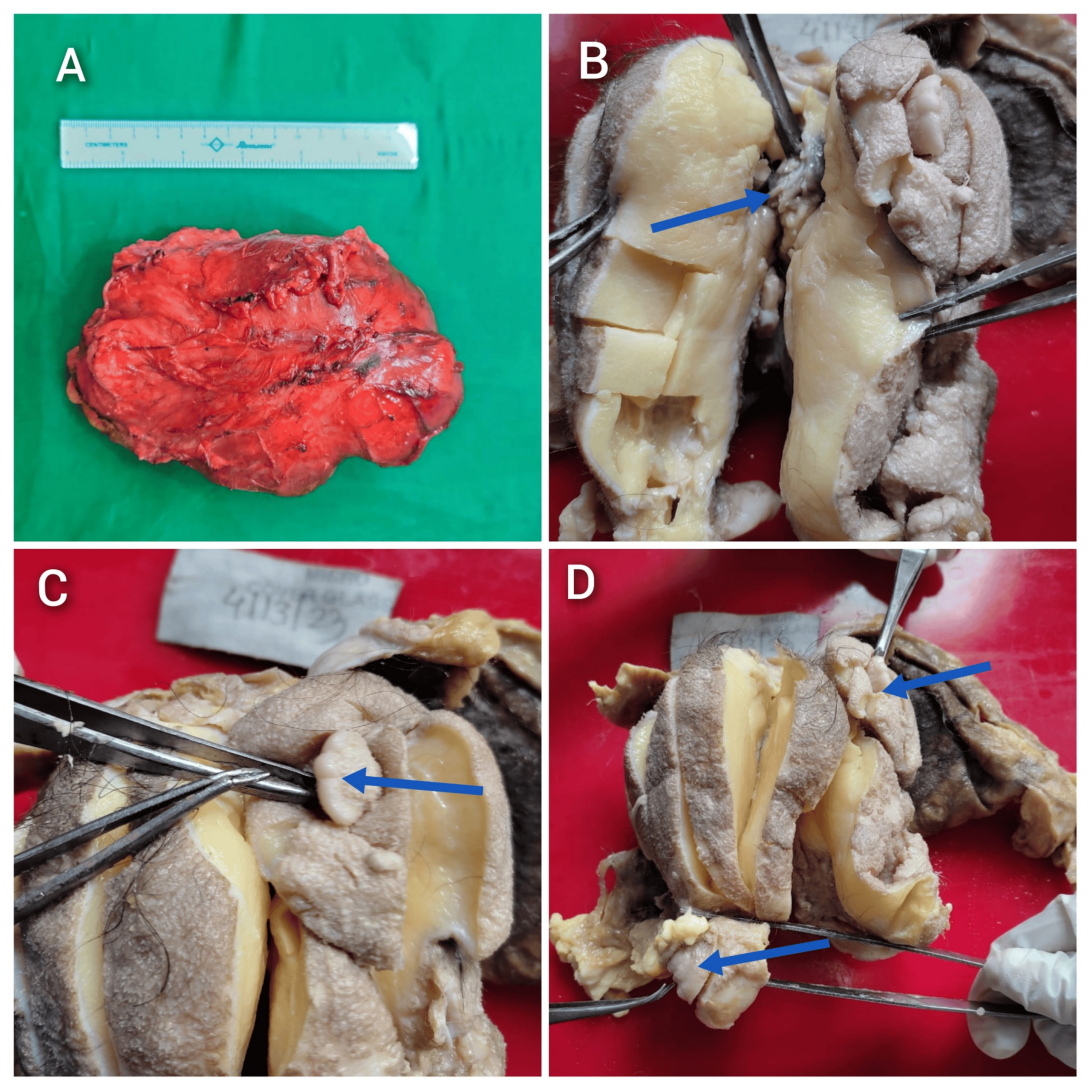
(A) Gross specimen. (B-D) Cut sections of the specimen (A) An irregular mass measuring 14×11×5 cm with a variably thickened surface. (B) The arrow indicates a centrally placed vertebral structure. (C) The arrow points to a well-developed limb-like structure. (D) The arrows highlight two limb-like structures.

On laparotomy, a retroperitoneal mass measuring 14×10 cm was observed, located in the left lumbar and iliac fossa. The mass, encased in a sac, was adherent to the left ureter and aorta. It displaced surrounding viscera without invasion of surrounding organs. The mass was excised in its entirety. Gross examination revealed an irregular mass measuring 14×11×5 cm, with a variably thickened surface covered in hair. The mass was irregular and yellowish-white and exhibited two ill-formed limb-like projections. Upon sectioning, a bony structure resembling vertebrae was visible at the center, flanked by conical structures. The central mass was yellowish-white with areas of cyst formation. The patient's postoperative period was uneventful (Figure 2).

**Figure 3:**
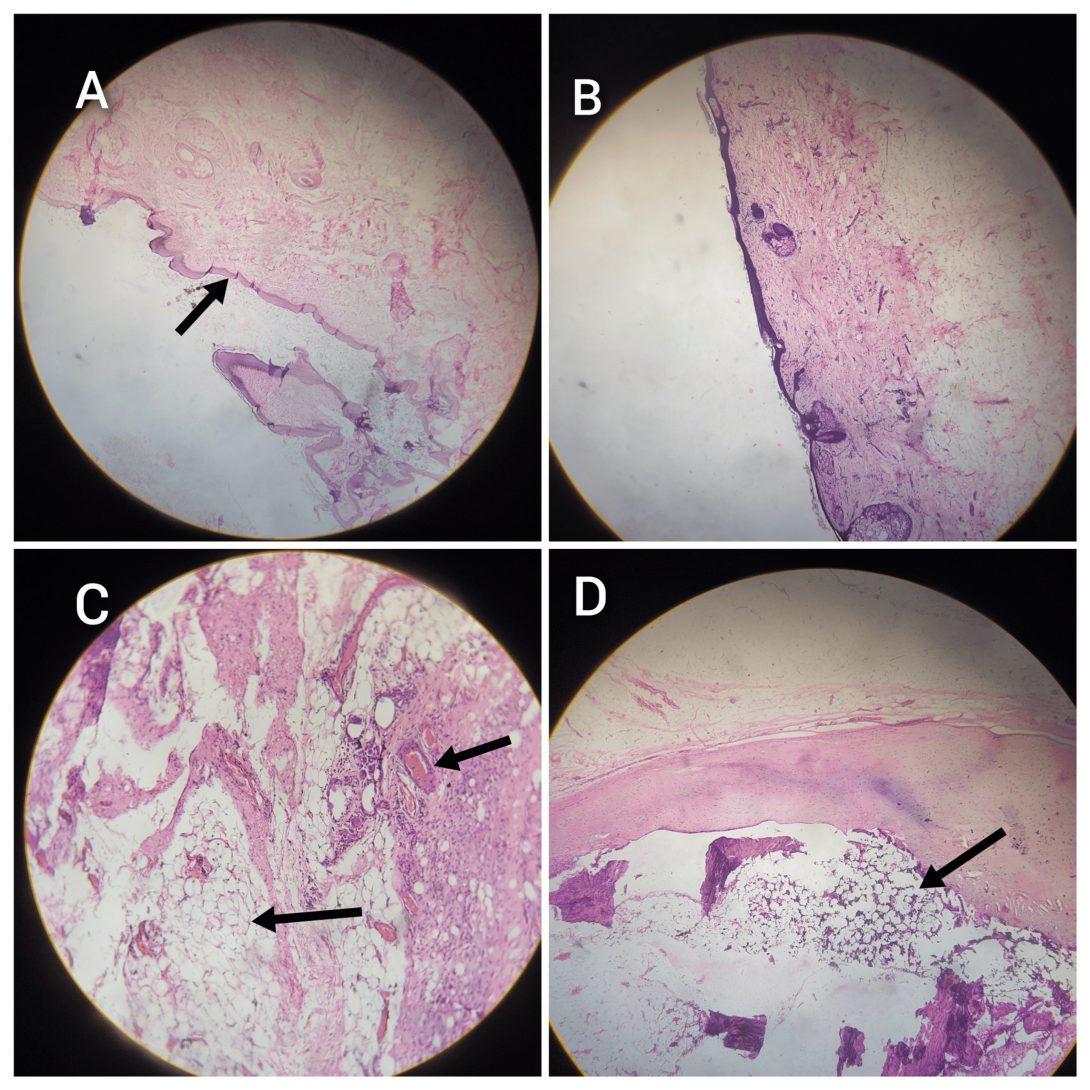
Histopathological sections (A) The arrow points to the outer wall of the mass, which shows stratified squamous epithelium and adnexal structures. (B) Well-formed mesenchymal structures. (C) The arrows highlight mature adipose tissue, blood vessels, and nerve bundles. (D) The arrow indicates a centrally located, well-developed bone with marrow.

Histopathological examination revealed the covering membrane consisted of keratinized stratified squamous epithelium with underlying cysts lined by foreign body giant cells. Sections of the mass revealed keratinized stratified squamous epithelium overlying fibro-collagenous tissue, which contained adnexal structures, abundant mature adipose tissue, bone with marrow, lymphoid tissue, and nerve bundles. Given the presence of the mass since early childhood, the organized arrangement of tissues from all three germ layers, and the presence of limb-like and vertebra-like structures, it was suggestive of FIF. On follow-up at 12 months, the patient is asymptomatic with normal β-HCG and AFP (Figure 3).

## Discussion

Several theories have been proposed to explain the pathogenesis of FIF. One widely accepted theory involves abnormalities during embryogenesis in monozygotic diamniotic twinning, where one fetus is encased within the host fetus due to the ventral folding of the trilaminar embryonic disc [3]. The parasitic twin is typically anencephalic and lacks many internal organs, often resulting in death before birth [4]. The initial growth of FIF mirrors that of twins but typically ceases abruptly due to one of two factors: either the vascular advantage of the host twin or an inherent pathology in the parasitic twin. The most common location for FIF is the retroperitoneum, accounting for 80% of cases [5]. However, FIF can also occur in rarer sites such as the oral cavity, scrotum, and sacrococcygeal region [6]. Another theory suggests that FIF may be part of the teratoma spectrum, with FIF representing a highly organized form of teratoma [7].

The primary entity to differentiate from FIF is an organized teratoma. Willis suggests that the presence of an axial skeleton, including a vertebral column, is a key criterion for distinguishing FIF from a teratoma [8]. FIF typically originates from a zygote during the primitive streak stage. The presence of vertebral elements indicates that gastrulation has progressed, as the fetiform mass develops to a level comparable to normal fetal development [9]. Many cases have been reported as FIF despite the absence of a vertebral column, but they exhibit features characteristic of advanced stages of organogenesis [10].

Most reported cases of FIF occur in infancy and the neonatal period, making our case unusual. Only nine adult cases of FIF have been documented in the literature [6,11,12]. Among these adult cases, the oldest was 47 years old and the youngest was 20 years old, whereas our case involves a patient who is 16 years old. Plain abdominal X-rays can assist in diagnosing issues with the vertebral column and axial skeleton. However, CECT and MRI are more effective for detecting the axial skeleton and the surrounding fatty tissue, as seen in our case [13]. Complete excision of FIF, including all surrounding membranes, is the treatment of choice due to the risk of malignant transformation [14,15]. Although FIF generally has a more favorable prognosis than cystic teratomas, the presence of immature elements necessitates thorough clinical, radiological, and serological follow-up to detect any potential recurrence.

## Conclusions

FIF is an extremely rare condition that presents as an abdominal mass in adulthood. It can be diagnosed before surgery, and complete excision of the mass is both curative and confirmatory. Although uncommon, it should be considered as a differential diagnosis for abdominal lumps in adults and must be carefully distinguished from teratomas, a more common variant.
